# Exploring Bacterial Microcompartments in the Acetogenic Bacterium *Acetobacterium woodii*

**DOI:** 10.3389/fmicb.2020.593467

**Published:** 2020-10-15

**Authors:** Nilanjan Pal Chowdhury, Lydia Alberti, Mark Linder, Volker Müller

**Affiliations:** ^1^Department of Molecular Microbiology & Bioenergetics, Institute of Molecular Biosciences, Johann Wolfgang Goethe University, Frankfurt, Germany; ^2^Max Planck Institute of Biophysics, Frankfurt, Germany

**Keywords:** propanediol utilization, anaerobic metabolism, acetogens, fermentation, bioengineering, gene expression, bacterial microcompartment

## Abstract

The strictly anaerobic acetogenic bacterium *Acetobacterium woodii* is metabolically diverse and grows on variety of substrates which includes H_2_ + CO_2_, sugars, alcohols and diols. It is unique in producing bacterial microcompartments (BMC) during growth on different substrates such as 1,2-propanediol, 2,3-butanediol, ethanol or fructose. In this study, we analyzed the genetic organization and expression of the BMC genes within the *A. woodii* genome, the previously described 18 gene *pdu* cluster as well as four other cluster potentially encoding one or two shell proteins. Expression analysis of respective gene clusters revealed that the *pdu* gene cluster is highly expressed during growth on 1,2-PD, 2,3-BD, ethanol and ethylene glycol. The promoter region upstream of the *pduA* gene was identified and used to establish a reporter gene assay based on chloramphenicol acetyl transferase as a reporter protein. The reporter gene assay confirmed the qPCR data and demonstrated that 1,2-PD is superior over ethanol and ethylene glycol as inducer. BMCs were enriched from cells grown on 2,3- BD and 1,2-PD and shown to have typical structure in electron micrographs. Biochemical analyses revealed several of the protein encoded by the *pdu* cluster to be part of the isolated BMCs. These data demonstrate a very unique situation in *A. woodii* in which apparently one BMC gene cluster in expressed during growth on different substrates.

## Introduction

Although bacteria are unicellular, some have developed specialized “organelles” such as bacterial microcompartments (BMCs). These BMCs harbor specialized metabolic enzymes for fast processing of toxic or volatile metabolic intermediates or increasing substrate concentration for enhancing metabolic flux (Bobik, 2006; Cheng et al., 2008; Kerfeld et al., 2010; Chowdhury et al., 2014). Being solely composed of selectively permeable proteins, they allow cross talk of their inner catalytic core to the cytosolic milieu thus allowing recycling of essential cofactors (Kerfeld et al., 2018). Depending on their functionality, BMCs are essentially divided into two major groups or classes, anabolic (carboxysome) or catabolic (metabolosome) (Kerfeld et al., 2005; Axen et al., 2014). Anabolic BMCs from cyanobacteria play an important role in global autotrophic CO_2_ fixation by carbon concentrating mechanism within their lumen and CO_2_ fixation by the encapsulated enzymes like RuBisCO and carbonic anhydrase (Shively et al., 1973). The catabolic metabolosomes involved in propanediol utilization (Pdu) or ethanolamine utilization (Eut) house substrate-specific signature enzymes like propanediol dehydratase or ethanolamine ammonia lyase that degrade their corresponding substrates to generate an aldehyde. This aldehyde is further disproportionated by aldehyde-processing enzymes to the corresponding alcohol and carbonic acids (Rondon and Escalante-Semerena, 1992; Penrod and Roth, 2006). Extensive studies have been undertaken to characterize the metabolic core and structural components of these BMCs. Irrespective of the source, BMCs share homologous structural proteins of mainly three types, BMC-H (hexamers), BMC-T (trimers) and BMC-P (pentamers) but different internal proteins (enzymes) (Klein et al., 2009; Kerfeld et al., 2018). Co-expression of all three structural genes (one BMC-H, one BMC-P and three BMC-T paralogs) from *Haliangium ochraceum* BMC in heterologous systems resulted in self-assembly of the proteins into an icosahedral shell with a diameter of 400 Å. In a path breaking effort, the crystal structure of this intact BMC shell has been recently published (Sutter et al., 2017).

In enteric and soil-dwelling bacteria like *Salmonella*, *Klebsiella*, *Shigella*, *Listeria*, *Yersinia* or *Lactobacillus* the genes for the structural proteins and the internal enzymes for BMCs are encoded within a single contiguous genetic cluster or locus (Jorda et al., 2013; Chowdhury et al., 2014). A bacterial genome wide survey has led to the discovery of BMC loci encoded in 23 bacterial phyla and distributed over 30 distinct locus subtypes (Jorda et al., 2013; Axen et al., 2014). One such subtype, propanediol-utilizing BMC from *Salmonella enterica* has been studied intensively over these years. The twenty-one *pdu* gene cluster which includes proteins and enzymes and two genes for transcriptional regulation (*pocR*) and a propanediol facilitator (*pduF*) is transcribed with all shell (*pduA, B, B’, J, K, N, T*, and *pduU*) and metabolic (*pduCDE*, *pduL*,*pduP*, *pduQ*, and *pduW*) genes when grown on 1,2-PD. It is noteworthy that most of the organisms studied so far have one BMC for one metabolic scenario. For example, the *pdu* gene cluster in *S. enterica* is only expressed when cells are grown on 1,2-PD either under oxic or anoxic conditions (Jeter, 1990; Bobik et al., 1997). However, a study from aerobic planctomycetes (*Planctomyces limnophilus*) has been shown to produce BMCs in response to growth on two different deoxy sugars, L-fucose and L-rhamnose (Erbilgin et al., 2014), where a single type BMC is utilized to house the toxic intermediate, lactaldehyde. Depending on the species, the BMC locus of the planctomycete generally contain 11–13 genes related to the metabolosome.

**FIGURE 1:**
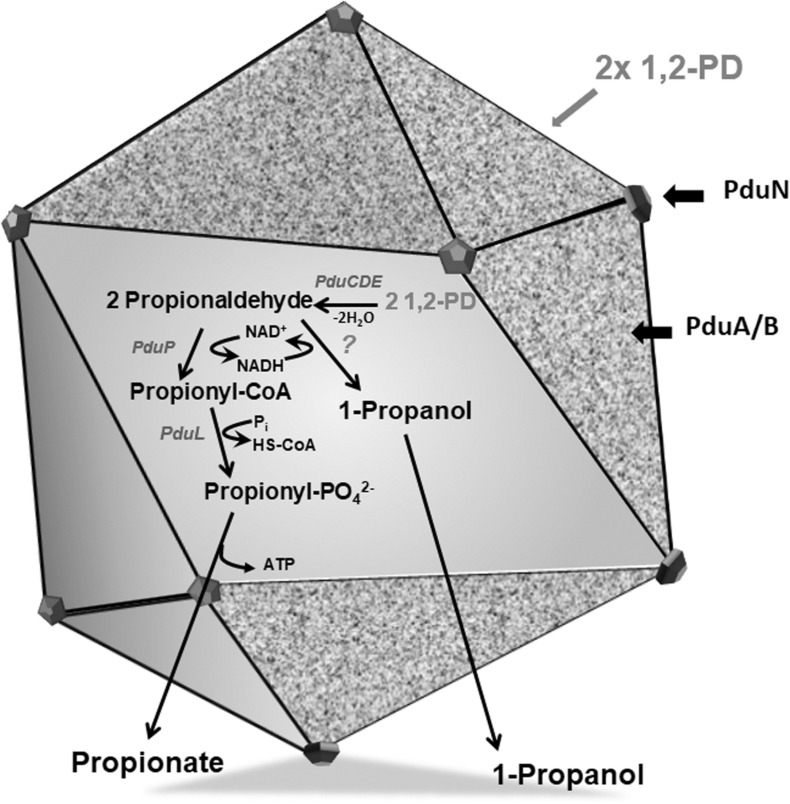
Model of a BMC encasing 1,2-PD metabolic pathway. The Pdu BMC encapsulates the signature enzyme PduCDE that dehydrates 1,2-PD to propionaldehyde which is further disproportionated to propionyl-CoA by PduP or propanol by an alcohol dehydrogenase, while NAD^+^/NADH is recycled by these two reactions. The proposed function of BMC is to sequester propionaldehyde within its lumen to avoid toxicity.

A very similar gene cluster is also found in the metabolically versatile, strictly anaerobic acetogenic bacterium *Acetobacterium woodii* (Schuchmann et al., 2015). The bacterium grows both chemolithoautotrophically, solely on hydrogen and carbon dioxide by utilizing the Wood-Ljungdahl pathway or chemoorganoheterotrophically on different substrates such as sugars, acids, alcohols, aldehydes or even amino acids (Drake, 1994; Müller, 2003; Ragsdale, 2008). In an earlier study, with growing and resting cells of *A. woodii*, we have shown that 1,2-PD is metabolized without involving acetogenesis by a disproportionation of propionaldehyde to propionate and propanol (Schuchmann et al., 2015). Genome wide analyses lead to the identification of the 20-gene cluster (Awo_c25930 to Awo_c25740) that encoded for the proteins responsible for 1,2-PD metabolism as well as BMCs (PduABB’KNT). Pairwise alignment of each gene of these 20 genes revealed strong similarity to the products of *pdu* gene cluster *S. enterica* (Schuchmann et al., 2015). As expected, BMCs were produced during growth of *A. woodii* on 1,2-PD and a hypothetical pathway for 1,2-PD utilization and its compartmentalization is shown in Figure 1. Unexpectedly, BMCs were also produced during growth on other substrates such as fructose, H_2_ + CO_2_, 2,3-butanediol, ethylene glycol or ethanol. This raised the question about the substrate specificity and induction of the BMCs and whether or not different BMC variants are produced during growth on different substrates. Here, we address the BMC gene organization in *A. woodii* and its transcriptional response to different growth substrates. Further, with enriched BMCs we show the protein profiling associated with BMC formation in the acetogen, *A. woodii.*

## Results

### *A. woodii* Has Five Islands Potentially Encoding Several Different BMC Shell Proteins

**FIGURE 2:**
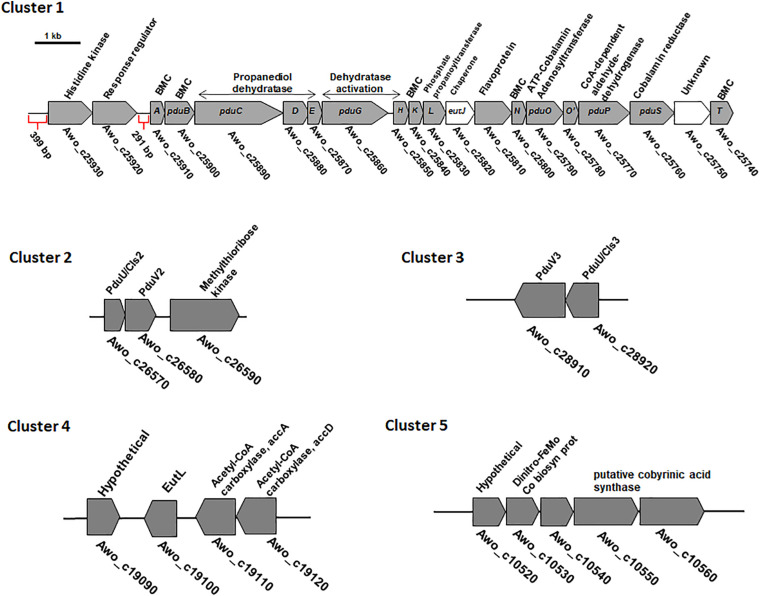
Genetic organization of different BMC gene containing cluster in the genome of *A. woodii*. The genes involved in 1,2-PD degradation and shell components clustered in a single continuous locus. The 18 gene *pdu* cluster is preceded by a two-component system consisting of histidine kinase and response regulator (Schuchmann et al., 2015). The locus encodes five structural shell proteins and internal proteins. A, *pduA*; E, *pduE*; H, *pduH*; K, *pduK*;M, *pduM*; N, *pduN*; T, *pduT*; U, *pduU*; V, *pduV*; BMC, bacterial microcompartment. In cluster 2 and 3, a BMC gene encoding PduU is clustered with a gene potentially encoding an acetate kinase. Cluster 4 contains a single pfam 00936 containing *eutL* gene clustered with acetyl-CoA carboxylase genes. Cluster 5 also contains a single pfam 00936 containing hypothetical gene which is clustered with genes encoding a putative cobyrinic acid a, c-diamide synthase.

The previously described *pdu* cluster in *A. woodii* consists of 20 BMC-associated genes, including a two-component system (Awo_c25930 to Awo_c25740) (Figure 2). In sharp contrast to *Salmonella enterica pdu* cluster, several BMC structural genes such as *pduJ*, *pduM* and *pduU* are apparently absent in the *pdu* cluster. While *pduA* and *pduK* encode hexameric shell proteins, *pduB* and *pduT* encode pseudohexameric shell protein and *pduN* codes for the pentameric cap protein for formation of BMC shell (Figure 1). It is noteworthy that in *Salmonella*, both PduJ and PduM contributes to the formation of normal shaped BMC and are thought to be essential for structural integrity of BMCs (Sinha et al., 2012; Chowdhury et al., 2016). However, a standard pfam based (Pfam00936/Pfam03319) BMC domain search across the *A. woodii* genome resulted in the identification of four more clusters that could potentially encode BMC proteins (Table 1). For simplicity, the *pdu* cluster (Awo_c25930 to Awo_c25740) is numbered as cluster 1 and the other gene loci as cluster 2–5, respectively (Figure 2). A pairwise gene comparison of *A. woodii* cluster 1 to that of *S. enterica pdu* cluster revealed strong resemblance of these two *pdu* clusters (Schuchmann et al., 2015). Cluster 2 consists of two genes, *pduU* and *pduV2* (Awo_c26570-80) (Figure 2). The gene Awo_c26570 (351 bp) encodes a shell protein, 63% identical to PduU from *Salmonella*. PduU are known to form hexamers, however, crystal structures of the low abundant PduU revealed that they do not form well-packed hexagonal layers and also have an occluded central pore (Crowley et al., 2008). The next gene is Awo_c26580 (468 bp) annotated as *pduV2* that encodes a protein of 17.4 kDa with 31% similarity to EutP from *S. enterica.* EutP has been recently reported to function as a novel acetate kinase (Moore and Escalante-Semerena, 2016). However, the *A. woodii* genome encodes an acetate kinase (Awo_c21260), *AckA* (1191 bp, 42.5 kDa) which is a considerably larger protein and involved in generation of acetate from acetyl-phosphate in the Wood-Ljungdahl pathway (Eden and Fuchs, 1983; Schuchmann and Müller, 2014). While both *pduU* and *pduV* genes are a part of the *S. enterica* 21-gene BMC cluster, these two genes are missing from cluster 1 in *A. woodii*. The next gene downstream of Awo_c26580 is methylthioribose kinase (Awo_c26590), unrelated to BMC function.

**TABLE 1:** Comparison of BMC proteins from *A. woodii* and *S. enterica.*

**Cluster number**	**Accession number/Gene name**	**Length (bp)/Predicted mass (kDa)**	**Pfam domain no.**	**Indentity^a^ (%) to *S. enterica* protein**
1	*pduA* (Awo_c25910)	276/9.1	00936	84
1	*pduB* (Awo_c25900)	786/26.9	00936	62
1	*pduK* (Awo_c25840)	363/12.5	00936	28
1	*pduN* (Awo_c25800)	279/9.7	00319	46
1	*pduT* (Awo_c25740)	528/18.3	00936	37
2	*pduU* (Awo_c26570)	351/29.5	00936	63
3	*pduU* (Awo_c28910)	375/13.3	00936	51
4	*EutL* (Awo_c19100)	645/23	00936	36
5	Hypothetical (Awo_c10540)	306/10.6	00936	-

*^*a*^Protein-protein identity was performed with BLASTp algorithm.*

Similar to cluster 2, cluster 3 also consist of the two genes *pduU* and *pduV3* (Awo_c28920-28910). While PduU encoded by cluster 2 is 55% identical to PduU from cluster 3, PduV2 (cluster 2) shares 37% identity to PduV3 (cluster 3). The next gene (Awo_c25900) downstream to the cluster 3 is a non-coding RNA gene, tRNA-Gly, unrelated to BMC formation.

The other two clusters, 4 – 5, comprise of single genes (Figure 2) with no other BMC related genes to its near proximity. Awo_c19100 (645 bp) codes for a protein similar to the *S. enterica* BMC shell protein, EutL (BMC-T). The nearest homolog of EutL in cluster 1 is PduB (25% identity). EutL is pseudohexameric (dimer of a trimer) and also the largest BMC domain protein in ethanolamine utilizing BMC (Sagermann et al., 2009). Interestingly, the gene Awo_c19100 was found to be localized as a single gene encoding a BMC-fold containing protein between a hypothetical protein encoding gene (in 3′ → 5′ direction) and downstream of acetyl-CoA carboxylase (acc) genes (in 5′ → 3′ direction). No promoter sequences could be predicted within the intergenic region (134 bp) of Awo_c19100 and *accA*.

The gene Awo_c10540 of cluster 5 encodes a protein that contains as single pfam00936 domain and a blast analysis of its predicted protein against protein database from *Salmonella* revealed a low sequence identity (<25%) to a putative inner membrane protein of *S. enterica* and no homologous protein could be found within the cluster 1. However, quite interestingly, a simple blast analysis of Awo_c10540 protein sequence against the entire protein database revealed a sequence similarity (45%) to a BMC-H type shell protein from *Clostridia*. A closer look into the protein revealed that the ‘so-called’ hypothetical protein is a close homolog of the unique sub-family of BMC protein, GrpU belonging to glycl-radical type BMC (GRM). Surprisingly Awo_c10540 is located in a cluster encoding a hypothetical protein, iron-molybdenum cofactor biosynthetic protein and a putative cobyrinic acid a, c-diamide synthase and no other BMC related genes could be identified in its near proximity. It is likely that the protein is involved in vit-B12 biosynthesis (Blanche et al., 1992). In sum-up, it is intriguing that multiple genes encoding components of BMC shell protein are distributed over different loci within the *A. woodii* genome. Therefore, it is important to study their role in BMC formation.

### Expression of *pdu* Gene Cluster Is Induced by Different Substrates

**FIGURE 3:**
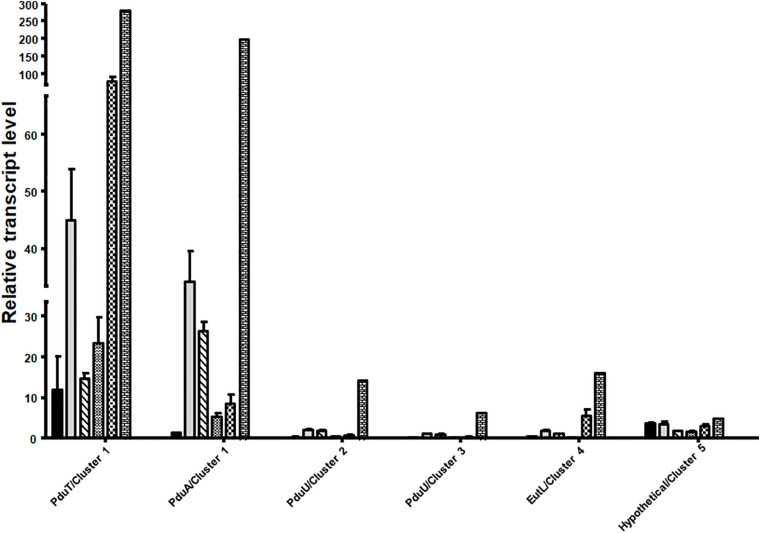
Differential regulation of the different BMC gene containing cluster in *A. woodii*. *A. woodii* was grown on 20 mM fructose, 20 mM ethylene glycol, 15 mM 1,2-PD, 20 mM 2,3-BD, 50 mM ethanol, or 5 mM acetaldehyde to early or mid-exponential phase. RNA was isolated from these cells and transcribed into cDNA and transcript levels were quantified by qPCR. Relative transcript levels of genes representative of each cluster, encoding PduT and PduA (cluster 1), PduU (cluster 2), PduU (cluster 3), EutL (cluster 4) or hypothetical (cluster 5) in *A. woodii* grown in fructose (black), ethylene glycol (gray), 1,2-PD (crossed), ethanol (small black dots), acetaldehyde (chequered), or 2,3-BD (bricks). Data are from three independent cultures and error bars represent one standard deviation.

Next, we analyzed mRNA levels of representative genes of each of the five clusters during growth on different substrates. qRT-PCR analyses were done with primers against the genes *pduT*/Awo_c25740 (cluster 1), *pduA*/Awo_c25910 (cluster1), Awo_c26570 (cluster 2), Awo_c28920 (cluster 3), Awo_c19100 (cluster 4) and Awo_c10540 (cluster 5). As shown in Figure 3, cluster 1 was by far the most expressed during growth on the substrates tested. Both *pduA* (first gene of cluster 1) and *pduT* (terminal gene of cluster 1) were expressed strongly. The transcript levels were up to 250 times higher than for the genes of the other cluster. Another comparison revealed a strong substrate dependence of transcript levels of both *pduT* and *pduA*, with the terminal gene in the operon having higher transcript levels than the first (also see Supplementary Figure S3). Expression of both *pduT* and *pduA* were highest during growth of 20 mM 2,3-BD while growth on 5 mM acetaldehyde led to second highest levels of *pduT* transcripts but levels of *pduA* did not follow this hierarchy. Ethylene glycol led to third-highest levels followed by ethanol and 1,2-PD. Astonishingly, transcript levels during growth on 1,2-PD were 76% and only 13% compared to cells grown on ethylene glycol and 2,3-BD, respectively. Although the transcript levels of genes of cluster 2, 3, 4, and 5 were at least 20–30 times lower in comparison to cluster 1, qRT-PCR analyses showed that 2,3-BD was the strongest inducer of the BMC genes.

### Identification of Promotor Regions Within the *pdu* Gene Cluster

**FIGURE 4:**
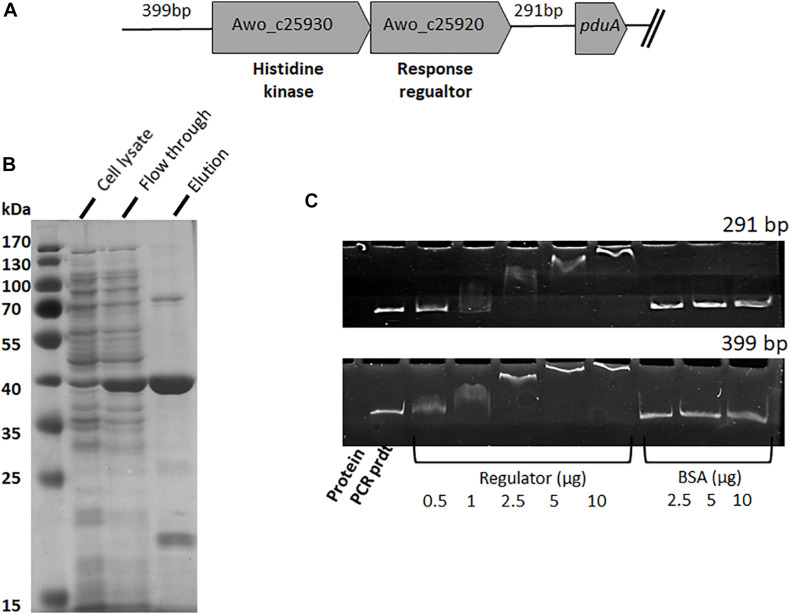
Binding of response regulator (Awo_c25920) to intergenic DNA of the *pdu* cluster. **(A)** Genetic organization of the histidine kinase and response regulator genes present upstream of the *pdu* gene cluster. Intergenic region upstream of his-kinase is 399 bp whereas downstream to the response regulator is 291 bp. **(B)** SDS-PAGE of purification steps of His 6-tagged response regulator, *Awo_c25920*, overproduced in *E. coli*. **(C)** EMSA performed with response regulator bound to two different intergenic regions. Shift assays contained 50 ng DNA template and increasing amount of regulator (0–10 μg protein), control assays received only DNA template and bovine serum albumin (2.5–10 μg protein). Samples were separated on a 6% native polyacrylamide gel, and the DNA was visualized with ethidium bromide.

To analyze the control and expression of the *pdu* gene cluster by different substrates, the promotor region(s) needed to be identified. Inspection of the genome sequence revealed two upstream regions, one upstream of *pduA* (Awo_c25910) (291 bp fragment) and one upstream of two-component sensor kinase (Awo_c25930) (399 bp fragment) that might contain the promotor regions (Figure 4A). To analyze whether these upstream DNA fragments bind the putative response regulator Awo_c25920, the gene was cloned into pET21a (+). A DNA sequence encoding a His-tag was inserted at the 5′-end of the gene and the plasmid was transformed into *E. coli* BL21 (DE3) (for protein sequence see Supplementary Figure S2). The expression of the regulator gene was induced with IPTG (1 mM) and the protein was purified from cell extracts over a Ni-nitrilotriacetic acid column using the hexa-histidine tag. The 43-kDa protein was purified to 95% homogeneity (Figure 4B). In addition, the 291 and 399-bp-fragments were amplified by PCR and purified. Next, we assessed *via* an electromobility shift assay, whether the response regulator binds one or both DNA fragments. In principle if the regulator binds to DNA, with increasing concentration of the regulator, the migration of DNA is retarded. Indeed, preincubation of increasing concentration of regulator (His tagged) (0–10 μg) with the 291 and 399-bp fragments (50 ng) resulted in a mobility shift of both the DNA fragments. In the assay where the response regulator protein was omitted or when bovine serum albumin was used instead of the regulator protein, electrophoretic mobility shift was not observed (Figure 4C). These results show that the response regulator protein binds specifically to the upstream region of Awo_c25930 and *pduA* indicating that the response regulator regulates expression of the *pdu* cluster and also its own gene.

### Generation of Reporter Genes to Analyze Promotor Specificity

To analyze substrate specificity for induction of *pdu* gene expression, the 291-bp fragment was cloned in front of a promotor-less *cat* (chloramphenicol acetyl transferase) gene. The construct was verified by DNA sequencing and transformed into *A. woodii*. Using this strategy, we aimed to control the expression of the *catP* gene under the transcriptional control of *pdu* promoter (291 bp) when grown on different growth substrates, as tested in this study. The induction of the *catP* gene was tested by measuring the CAT enzyme activity. For the following induction experiment, a carbon source for growth of *A. woodii* was required that did not induce BMC formation. To test whether BMCs were induced, the cellular levels of the putative BMC protein (PduB) was immunologically tested with anti-PduB antibodies in cell extracts prepared from *A. woodii* cells grown on 2,3-BD (20 mM), lactate (50 mM), formate (100 mM) or methanol (60 mM) as earlier described (Schuchmann et al., 2015). PduB was present in cells grown on 2,3-BD and lactate but not in methanol or formate-grown cells (Supplementary Figure S4). Therefore, 100 mM formate was used as carbon and energy source for the following experiments.

**FIGURE 5:**
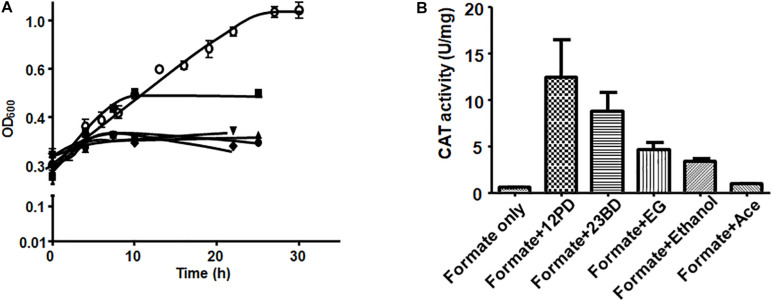
Substrate-dependence of *pdu* promoter activity. **(A)** Growth of *A. woodii* harboring plasmid pMTL82254-P_pduA_ on addition of respective substrate after growth on 100 mM formate (•), 15 mM 1,2-PD (■), 20 mM 2,3-BD (▲), 50 mM ethylene gycol (▼), 5 mM acetaldehyde (◆), 50 mM ethanol (○). Time zero correspond to late exponential growth phase on 100 mM formate where induction was done. **(B)** CAT activity of *A. woodii* harboring plasmid pMTL82254-P_pduA_, induced by different substrates. Formate only, corresponds to the culture without inducing substrate added.

For the reporter (*catP*) gene assays, *A. woodii* cells harboring the reporter plasmid (291bpUp_pMTL82254) were first grown on formate as sole carbon and energy source to late exponential or early stationary phase and then *catP* expression was induced by addition of different substrates such as 1,2 PD (15 mM), 2,3-BD (20 mM), ethylene glycol (50 mM), acetaldehyde (5 mM) or ethanol (50 mM). Growth was monitored for the first 10 h post induction and cells were harvested after 24 h. After addition of 1,2-PD, cells doubled once in 10 h and then the OD_600_ stayed constant in the next 24 hr. With ethanol as a substrate, the cells doubled twice to reach a final OD_600_ of 1.0. No diauxic growth or adaptation (lag phase) time was observed when *A. woodii* cells were shifted from formate to 1,2-PD or ethanol. Addition of 2,3-BD, ethylene glycol or acetaldehyde led to only a marginal increase in OD_600_ during 24 h post induction (Figure 5A).

CAT assays were performed with cell lysates. As expected, in case where no inducer substrate was added (formate only), no CAT activity could be observed. Induction by 1,2-PD led to highest CAT activity of around 12.4 U/mg followed by 2,3-BD (8.7 U/mg), ethylene glycol (4.6 U/mg) and ethanol (3.3 U/mg). However, with acetaldehyde as an inducer, CAT activity was only 7.5% of the maximal activity (0.94 U/mg) indicating very low inducing power of acetaldehyde (Figure 5B).

### Enriched Microcompartment Reveals Globular Shaped Particles

**FIGURE 6:**
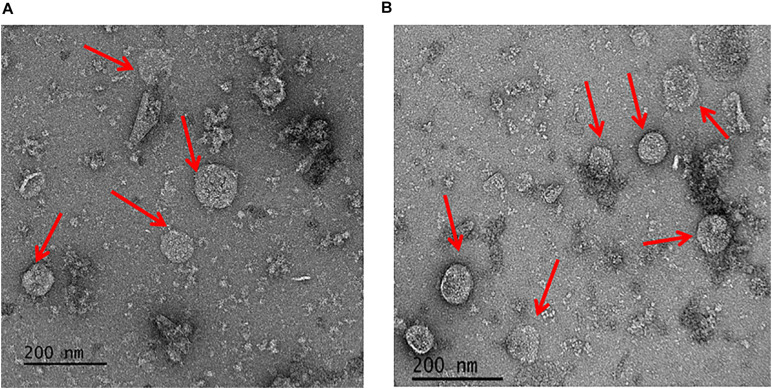
Electron micrographs of enriched BMCs. Isolated BMC particles from *A. woodii* cells grown on 1,2-PD **(A)** or from 2,3-BD **(B)**. *Arrowheads* point to BMCs, size varied from 60–140 nm (particles number, *n* > 100).

**FIGURE 7:**
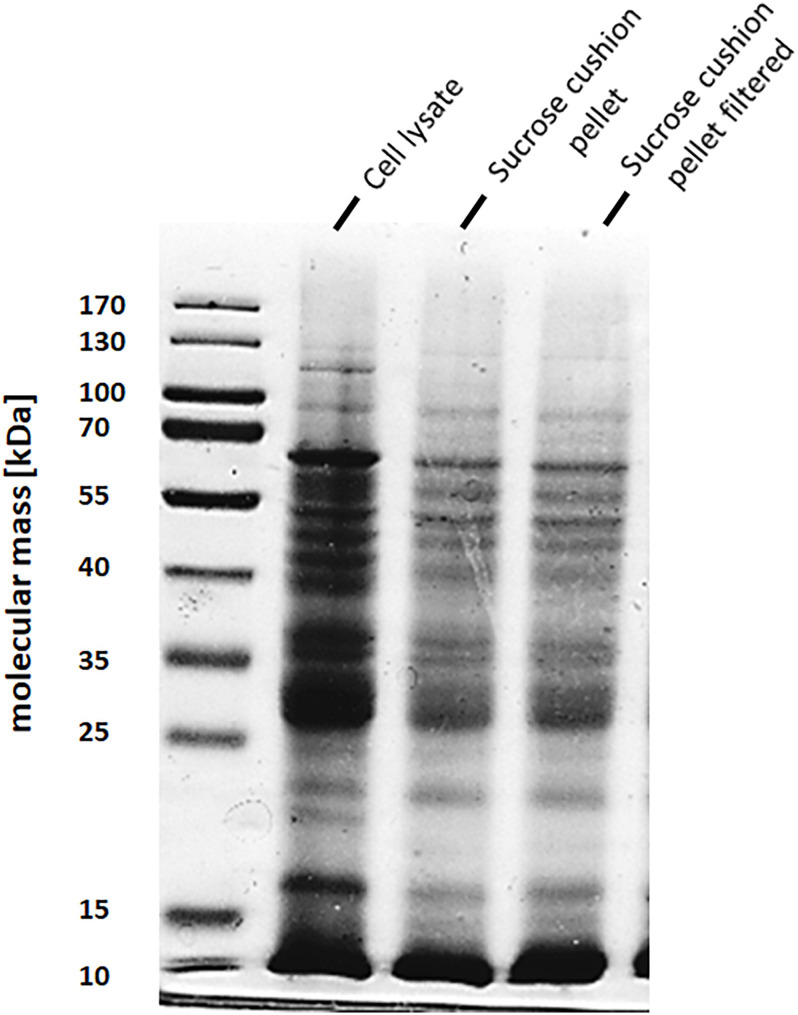
SDS-PAGE of enriched BMC fraction. BMCs from 2,3-BD or 1,2 PD cells were separated on a 12% denaturing polyacrylamide gel (Laemmli). A *Lane 1*, molecular mass standards; *lane 2*, sucrose cushion pellet fraction, lane 3, 0.45 μm filtered pellet resuspension fraction (enriched BMCs).

**FIGURE 8:**
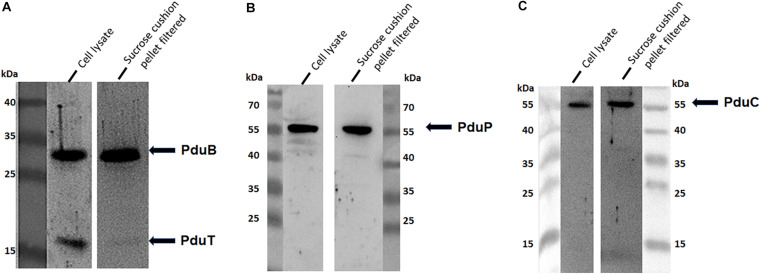
Immunological detection of key components of BMC in cells grown on 2,3-BD. Enriched BMC fractions were separated on a 12% SDS-polyacrylamide gel. The presence PduB and PduT **(A)**, PduP **(B)**, and PduC **(C)** was determined immunologically in both cell lysates and enriched fractions.

To address the nature of the proteins comprising the BMCs, they were enriched from *A. woodii* cells. Therefore, cells were grown on 1,2-PD or 2,3-BD to an OD_600_ of 0.25 and 0.6, respectively and BMCs were enriched from the prepared cell lysates as described in the methods section. The cloudy protein solution after resuspension of the BMC pellet was cleared twice by centrifugation (10000 × g, 20 min, 4°C) and the enriched BMC fraction (20 μg/ml) was immediately mounted on copper grids and negatively stained with 1% uranyl formate. Electron micrographs revealed distinct globular shaped bodies ranging from 60–140 nm in size (Figure 6). The majority of the particles were ruptured and particulate debris were found clustered around the intact BMCs. The BMCs were highly unstable and mostly lost its integrity during preparation. To further confirm that the enriched globular structures actually represent BMCs, the enriched fractions were separated by PAGE. At least 16 proteins were visible after staining with Coomassie blue (Figure 7). Further, the presence of the putative BMC shell and internal protein were analyzed with polyclonal antisera raised against heterologously produced PduB or PduT (shell protein) and PduP (CoA-dependent propionaldehyde dehydrogenase) or PduC (propanediol dehydratase large subunit) as mentioned earlier (Trifunović et al., 2016). As can be seen in Figure 8, the enriched preparation contained both shell proteins PduB and PduT along with internal proteins PduP and PduC. The detection of PduB and PduT were done on the same blot by incubating the membrane with anti-PduB and anti-PduT antibodies, no cross reactivity of PduB and PduT was observed. The enriched BMC fractions after trypsin digestion were subjected to mass spectrometric analysis. This resulted in detection of several gene products from cluster 1. The major shell proteins that were detected were PduA, PduB, PduK and PduN. PduT could not be detected in the mass spectrometric analysis due to its low abundance in BMC shells. No gene products or shell proteins encoded by cluster 2,3, 4 or 5 could be detected, which also substantiates our earlier results.

## Discussion

Acetogenesis involves a cascade of both soluble and membrane proteins for harvesting electrons either from hydrogen or organic substrates and channels them to product formation in form of acetate, ethanol or butyrate (Müller et al., 2004). Uniquely, the versatile bacterium *A. woodii* grows heterotrophically on variety of substrates like hexoses (fructose), diols like 2,3-butanediol (Hess et al., 2015) or ethylene glycol (Trifunović et al., 2016) and ethanol (Buschhorn et al., 1989; Bertsch et al., 2016) as sole carbon source for growth. In each case it employs a variety of distinct protein complexes involved in catabolism of these substrates. Most importantly, *A. woodii* can also undergo a non-acetogenic mode of lifestyle during growth on 1,2-PD as a sole carbon and energy source and produces almost equal amounts of propionate and propanol as catabolic end products (Schuchmann et al., 2015). Coenzyme B12-dependent growth on 1,2-PD in *S. enterica* and other enteropathogens such as *Shigella*, *Klebsiella*, *Yersinia* and *Listeria* or *Lactobacillus* is known to occur within specialized proteinaceous bacterial microcompartments with loaded metabolic enzymes within its core (Toraya et al., 1979; Cheng et al., 2008). These bacteria involve BMCs not only to optimize metabolic segments of 1,2-PD metabolism but also to sequester the toxic metabolic intermediates like acetaldehyde/propionaldehyde (Sampson and Bobik, 2008). In case of *A. woodii*, metabolism of diols and ethanol proceeds *via* the formation of acetaldehyde as a metabolic intermediate and hence BMCs are formed during growth on these substrates. In our previous study with *A. woodii*, we described the metabolic scheme of 1,2-PD and showed that this led to formation of bacterial microcompartment within the cells. We found that genes encoding the metabolic enzymes involved in 1,2-PD catabolism co-localizes with BMC shell component genes within a single BMC gene cluster within *A. woodii* genome (Schuchmann et al., 2015).

In this study, we have shown that the genome of *A. woodii* harbors five different gene clusters that potentially encodes BMC shell but the major *pdu* cluster (cluster 1) is responsible for encoding proteins for BMC formation. While Awo_c25740 (*pduT*), Awo_c25840 (*pduK*), Awo_c25900 (*pduB*) and Awo_c25910 (*pduA*) belongs to the cluster 1, the rest of the four putative BMC shell protein encoding genes are distributed over four gene loci (clusters 2–5). Cluster 1 genes responsible for shell assembly like *pduA* and *pduK* (hexamer), *pduN* (pentamer) and *pduB* and *pduT* (trimer) were strongly expressed in *A. woodii* during growth on diols and ethanol. In contrast, Awo_c26570 (cluster 2) and Awo_c28920 (cluster 3) that code for the BMC shell protein, PduU, an integral part of the BMC cluster in a related acetogen, *A. longum* (Tocheva et al., 2014) or enteropathogenic *S. enterica* (Bobik et al., 1999), did not exhibit similar high levels of *pduU* expression in *A. woodii* during growth on the substrates tested. Interestingly, *pduU* which is an integral part of the *pdu* operon in *A. longum* and *Salmonella*, is absent from *A. woodii pdu* cluster 1. The positioning of *pduU* genes along with potential acetate kinase genes at multiple sites within the *A. woodii* genome suggested that these two gene clusters are only expressed during growth on specific substrates and each PduU shell protein permits the entry of a particular metabolite. The low transcript levels of these two clusters (20–30-fold) in comparison to cluster 1 clearly suggested that it is highly unlikely that the gene products of these two clusters play an important role in *A. woodii* BMC formation, at least during growth on the substrates tested here. In earlier studies, PduU has been reported to be a minor shell component and deletion of the gene did not greatly influence BMC structure or growth of *Salmonella* on 1,2-PD (Cheng et al., 2011). *S. enterica* with a *pduU* deletion grew slightly slower than the wild type (67% of maximal growth rate) but formed normal shaped BMC. Also, the crystal structure of PduU revealed that it contains a circularly permutated BMC domain fold with blocked pore and is predicted not to be involved in any transport processes which also corroborates our finding (Crowley et al., 2008). Similar to cluster 2 and 3, qRT-PCR experiments revealed low transcript levels of either cluster 4 or 5 during growth on the substrates tested. One interesting feature of cluster 4 is that it consists of a single gene encoding an EutL homolog, an essential component of ethanolamine utilizing BMC found in *Salmonella* (Kofoid et al., 1999) and *Escherichia coli* (Tanaka et al., 2010) where it plays an important role in substrate transport. However, the low expression of Awo_c19100 (*eutL*) during growth on different substrates Hints that this protein is unlikely to be involved in BMC formation and transport of the substrate tested. In Pdu BMC of *Salmonella* (Lehman et al., 2017) and *Lactobacillus* (Pang et al., 2012), the protein that exhibits homology to EutL is the shell protein PduB. In crystal structures of EutL (Sagermann et al., 2009) and PduB (Pang et al., 2012), a gated pore at the center of the protein trimer has been revealed. It is clear that the role EutL plays for Eut-BMC, PduB does for Pdu-BMC. While EutL and PduB are orthologs, PduB is the major architectural shell protein in Pdu metabolosome. In a similar scenario, PduB is also strongly expressed in *A. woodii*, during growth on the substrates we tested and possibly along with PduA forms shell architecture and controls multiple substrate transport into *A. woodii* BMC. It still remains unclear why a single gene (Awo_c19100) encoding EutL (cluster 4) is present downstream of acetyl-CoA carboxylase gene within *A. woodii* genome. Finally, cluster 5 that codes for a hypothetical protein is actually a homolog of GrpU from glycyl-radical microcompartment (GRM) from *Clostridia*. Similar to Pdu BMC, GRM function to encase enzymes for 1,2-PD metabolism with only an exception, instead of the signature enzyme PduCDE it encases a glycyl-radical type 1,2-propanediol dehydratase and its activating enzyme. GrpU which forms hexameric structure is known to be a highly divergent type of BMC shell protein and its crystal structure revealed to bind a Fe-S cluster at its center (Thompson et al., 2014). It has been proposed that GrpU either facilitates electron transport through shell of Grp BMC to generate the glycyl radical at the active site of the Grp-diol dehydratase or transport Fe-S cluster necessary for the function of the encapsulated dehydratase. However, in case of *A. woodii*, this GrpU like hypothetical BMC shell protein encoding gene is found clustered with genes encoding a putative cobyrinic acid-diamide synthase and a dinitrogenase iron-molybdenum cofactor biosynthetic protein and not any other BMC related genes. While it is have been found gene clusters encoding GRM or Pdu-BMC never co-occur in any genome (Zarzycki et al., 2015), it is unlikely that this hypothetical protein play an important role in BMC formation and likely in vitamin biosynthesi.

Studies from *S. enterica* showed that expression of the *pdu* operon was controlled by PocR in response to 1,2-PD (Chen et al., 1994, 1995; Rondon and Escalante-Semerena, 1996). The upstream region of the *pdu* cluster in *A. woodii* also encodes a similar two-component system (histidine kinase and response regulator) and our electrophoretic mobility shift assays revealed that indeed the regulator encoded by Awo_c25920 binds both to the upstream region of the *pduA* (first gene of the *pdu* cluster) and also to the upstream region of histidine kinase (Awo_c25930). This indicated that in contrast to *S. enterica*, a single regulator in *A. woodii* works in both cases. Using a reporter gene fused to the upstream region of the *pduA* gene (291 bp) we found induction of the reporter gene by 1,2-PD, 2,3-BD, ethylene glycol, ethanol and acetaldehyde (in order of decreasing intensity). 1,2-PD led to the highest induction (25-fold) of the reporter gene proving it to be the preferred natural carbon source for induction of BMC genes. However, either of the diols (2,3-BD or ethylene glycol) or ethanol also induced BMC gene expression. It is worth a mention, it is likely the promiscuity of the response regulator which possibly sense two or three-carbon alcohol (ethanol or diols) and induce BMC formation. While *A. woodii* also grow on methanol, they do not produce BMCs (see Supplementary Figure S4), most likely the regulator cannot sense single carbon alcohol. Interestingly, induction by acetaldehyde was mere 2-fold higher than in the control where no inducer was added (formate only). Since the first step in metabolism of acetaldehyde by *A. woodii* involves the key enzyme AdhE to produce ethanol and acetyl-CoA (Trifunovic et al., 2020), it might be the case that ethanol is the actual inducer of *pdu* operon during growth on acetaldehyde. Since the reporter gene assay was carried out in a 24 h time frame and no growth was observed on addition of acetaldehyde, possibly low ethanol formation resulted in low induction of the reporter gene.

The outcome of the reporter gene assay presented sharp contrasting results to our quantified BMC gene expression levels of *A. woodii* during growth on different substrates. qPCR experimental results revealed that 2,3-BD strongly the BMC gene expression in comparison to growth on 1,2-PD. While qPCR analysis quantifies relative abundance of RNA transcripts within the cell, this measurement is markedly influenced by multiple factors like rate and stages of growth. In most cases, quantified gene expression levels do not reflect the final quantity of protein product formed within the cell. *A. woodii* displays variable growth optima and phenotype depending on the carbon source available for growth. It grows to a final optical density of 0.65 when grown on 2,3-BD as a sole carbon source, on the other hand optimum growth on 1,2-PD was mere 40% (OD_600_ = 0.25) when compared to growth on 2,3-BD. Owing to different growth maxima of *A. woodii* on the carbon sources tested, a direct co-relation of gene expression to inducing power of a substrate directly from the qPCR experimental results would have been incorrect. However, a reporter gene CAT assay quantifies the specific activity as function of protein abundance, this provides a better measure of substrate induction power. This is what we expected, strongest induction by 1,2-PD and related diols (2,3-BD or ethylene glycol). Overall, our results indicate that the response regulator senses the respective diols or ethanol, binds to the *pdu* promoter to mediate gene expression and hence formation of BMCs.

In *A. woodii*, it is highly likely that only the shell proteins encoded by the *pdu* cluster constitutes the BMC shell, in which, mainly PduB and PduA allow preferential movement of substrates and product through the shell. However, one interesting aspect that needs further clarification is that though *pduT* is highly expressed in the in the growth conditions tested, it was never detected in mass-spectrometric analysis, though its presence could be detected by immunoblotting. We opine that due to low abundance of PduT in the final BMC shell, this protein was not detected by standard mass-spectrometric technique. This brings us to a further question; is the scenario same for other shell proteins from clusters 2–5 (though having a low transcript for each gene). In our knowledge this is likely not the case. However, since there is a limitation of detecting low abundant protein constituting BMC shells this question will remain open until protein specific antibodies are raised and probed against cell extracts of *A. woodii* grown on different carbon substrates.

Finally, we found that, BMCs enriched from 2,3-BD- or 1,2-PD-grown cells were less regular than carboxysomes purified from the marine cyanobacterium *Prochlorococcus marinus* MED4 (Roberts et al., 2012) For most BMC particles visualized (>100) under the electron microscope, instead of a polygonal symmetry, *A. woodii* BMCs appeared spherical, ranging from 60–140 nm. The shells were not all of same shape and size. In our experience, we found BMCs from *A. woodii* were extremely sensitive to handling and rapidly degraded over time. Overall, in this study we have shown that though *A. woodii* codes for multiple BMC shell proteins within its genome, most likely the 20 gene *pdu* cluster is solely responsible for formation of BMC during growth on different substrates. Finally, the question to how these different substrates are transported within the BMC remains wide open.

## Materials and Methods

### Growth of *A. woodii*

*Acetobacterium woodii* (DSM 1030) was grown at 30°C under anoxic conditions. The medium was prepared as described previously (Heise et al., 1989). Fructose (20 mM), 1,2-propanediol (15 mM), 2,3-butanediol (20 mM), acetaldehyde (5 mM), ethylene-glycol (20 mM) or ethanol (50 mM) served as carbon and energy source. Growth was determined by measuring the optical density at 600 nm (OD_600_).

### Preparation of RNA

For RNA isolation, *A. woodii* strains were subcultured at least twice on the indicated substrate after initial inoculation from the substrate-adapted stock culture. Cells were grown to the mid-or early exponential growth phase and harvested by centrifugation (10,000 × *g*, 10 min, 4° C). The cells were then frozen in liquid nitrogen and stored at −80°C until RNA extraction. Thawed cells were disrupted using a cell homogenizer (RETSCH GmbH, Germany), and RNA was purified with a RNeasy mini kits (QIAGEN, Hilden, Germany) according to the manufacturer’s instructions. To ensure complete removal of DNA, 10 μg nucleic acids were used for Turbo DNase treatment (Thermo-Fisher, MA, United States) in the presence of ribonuclease inhibitor, RNasin (Promega, Mannheim, Germany). The DNase digested RNA fraction was further purified with RNeasy mini kit. The quality of RNA (integrity and DNA contamination) was determined after separation on an agarose gel (1%, wt/vol, in 1 × TBE buffer containing 89 mM Tris–HCl, 89 mM boric acid and 1 mM EDTA). 1 μg of RNA from each sample was converted into total cDNA by using M-MLV Reverse Transcriptase according to the manufacturer’s protocol (Promega, Mannheim, Germany).

### Quantitative PCR (qPCR) Analysis

All quantitative PCRs were performed in triplicate, using SsoFast EvaGreen Supermix (Bio-Rad, CA, United States) with 1 ng cDNA as template and 500 nM of gene specific primers in a final reaction volume of 25 μl. Cycling parameters were set per optimized cycling conditions for a Rotor-gene RG-3000 qPCR cycler (Corbett Research, Cambridge, United Kingdom). Standard curves for quantification of transcripts were generated using dilutions of *A. woodii* chromosomal DNA from 1 × 10^–4^ ng to 10 ng. Transcript level of representative genes for *pdu* operon and 4 other gene cluster were analyzed using primers for Awo_c25740 (*pduT*), Awo_c25910 (*pduA*), Awo_c26570 (*pduU*), Awo_c28920 (*pduU*), Awo_c19100 (*eutL*). Transcript levels of all the genes tested were normalized to transcript levels of the gene *gyrA* which encodes the housekeeping enzyme gyrase. Forward and reverse primer used for each gene are listed in supporting information (Supplementary Figure S1).

### Cloning and Overproduction of Regulator

The response regulator gene, Awo_c25920 was amplified from chromosomal DNA of *A. woodii* with primers Reg_for and Reg_rev (Supplementary Figure S1). The PCR product was cloned in to pET-21a (+) expression vector and was transformed into BL-21 (DE3) for overproduction of the H_6_- tagged_regulator (Reg). The transformed cells were grown at 37°C in LB medium to an OD_600_ of 0.6–0.8 and gene expression was induced by addition of Isopropyl-D-galactopyranoside (IPTG) to a final concentration fo 1 mM. Cells were grown overnight at 16°C under shaking conditions. The overproduced protein was purified using 6X-Histidine tag (for protein sequence see, Supplementary Figure S2) from the cell lysates prepared from harvested cells using buffer A [50 mM sodium phosphate buffer (pH 8.0), 250 mM imidazole and 300 mM NaCl]. The protein purity was analyzed in a 12% denaturing polyacrylamide gel (Laemmli, 1970) and further staining with Coomassie Brilliant Blue G250 (Meyer and Lambert, 1965).

### Electromobility Shift Assays

Intergenic regions between the response regulator gene (Awo_c25920) and *pduA* (Awo_c25910) (291 base pairs) and upstream region (399 bp) of the histidine kinase gene (Awo_c25930) were amplified *via* PCR using chromosomal DNA of *A. woodii* as template. Primer pairs 291down_for/rev and 399up_for/rev used for PCR amplification are listed in supporting information. 500 μg of His-tagged response regulator was phosphorylated in buffer B (50 mM Tris–HCl, 125 mM NaCl, 10 mM glycerol, 5 mM MgCl2, pH 7.8) containing 100 mM acetyl-phosphate for 1 h at RT. DNA binding assays were performed in 12 μl of buffer B, by incubating 50 ng of each PCR product with 0–10 μg of phosphorylated protein. Control assays were performed using BSA (2.5–10 μg) as negative control. The samples were mixed with loading buffer [1X-TAE buffer, 10% (vol/vol) glycerol, bromophenol blue and xylene cyanol] and loaded onto a 6% native polyacrylamide gel. Electrophoresis was performed at 105 V in 1X TAE for first 90 min and then at 300 V till completion, the temperature was strictly maintained at 4°C. The gel was stained with ethidium bromide and DNA was visualized under a UV-transilluminator (INTAS Imaging, Göttingen, Germany).

### Reporter Gene Plasmid and Transformation

For construction of the reporter gene plasmid, 291 bp upstream intergenic region of *pduA* was amplified from *A. woodii* genomic DNA by PCR. The amplified fragment was fused upstream between the *Not*I and *Nde*I sites of the promoter-less *catP* reporter plasmid pMTL82254. The generated reporter gene construct is designated as 291bpUp_pMTL82254_CAT that allow the expression of CAT (chloramphenicol acetyl transferase) under the control of *pdu* promoter. This plasmid was routinely introduced into *A. woodii* cells according to earlier described procedure (Westphal et al., 2018). The transformed *A. woodii* cells were plated onto Heise agar medium containing 20 mM fructose and 15 μg/ml erythromycin. Single colonies were picked and transferred in 5 ml Heise medium containing 20 mM fructose and 15 μg/ml erythromycin. Cells were tested for plasmid insertion by PCR amplification using primers for 291 bp region and also *CAT* reporter gene.

### CAT Reporter Gene Assays

250 ml Heise medium containing 100 mM formate as carbon source and 15 μg/ml erythromycin was inoculated with a preculture of transformed *A. woodii* cells grown on 20 mM fructose and 15 μg/ml erythromycin. The reporter gene expression was induced with 15 mM 1,2-PD, 20 mM 2,3-BD, 50 mM ethanol, 5 mM acetaldehyde or 20 mM ethylene glycol in the early stationary growth phase on 100 mM formate, OD_600_ (0.25–0.3). Post induction change in growth was monitored for first 10 h and finally after 24 h. Induced cells were then harvested and resuspended in 500 μl of B-PER reagent (Thermo Fisher, MA, United States) and lysed using a cell homogenizer (RETSCH GmbH, Germany) for 5 min cycles, twice. Cell debris were removed by centrifugation (12,000 × *g*, 20 min) and the supernatant obtained was used for chloramphenicol acetyltransferase (CAT) assays (Shaw, 1975). Chloramphenicol acetyl transferase catalyze the transfer of acetyl moiety from acetyl-CoA to a chloramphenicol molecule to form chloramphenicol 3-acetate. The released CoA moiety then reacts with DTNB (5,50-dithiobis- 2-nitrobenzoic acid) to form yellow TNB (5-thio-2-itrobenzoic acid), the activity is measured by measuring the rate of TNB formation. The activity was measured in 1 ml quartz cuvettes containing 50 mM Tris buffer (pH 7.8) and 250 μM DTNB, 100 μM acetyl-CoA and 10 μl cell lysates. The reaction was initiated by addition of 10 μl of 0.3% w/v chloramphenicol. The formation of the yellow 5-thio-2-nitrobenzoic acid (TNB) was followed at 412 nm, ε = 13.6 mM^–1^cm^–1^ (Silverstein, 1975).

### Microcompartment Enrichment

*A. woodii* was grown on 1,2-PD or 2,3-BD and harvested as described earlier. 1 g wet cells were washed and re-suspended in buffer C (50 mM Tris–HCl, 420 mM sucrose, 0.1% 1,2-PD, 30 mg lysozyme, pH 8.0) and incubated for 1 h at 37°C. The cells were sedimented by centrifugation (10,000 × *g*, 10 min) and resuspended in 10 ml buffer D (50 mM Tris/HCl, 0.5 mM PMSF, 0.1 mg ml^–1^ DNAseI, pH 8.0). Cells were disrupted by passage through a French press (SLM Instruments, United States) at 100 MPa. Cell debris were removed by two centrifugation steps (8000 × *g*, 20 min). The supernatant (2 ml each) was collected and layered over a sucrose cushion (30% w/v sucrose in 50 mM Tris pH 8.0, 6 ml) and centrifuged in a TFT 65.13 rotor (49,000 × *g*, 16 h). The supernatant was discarded and the pellet obtained was washed and resuspended in 5 ml Tris pH 8.0, 0.1% 1,2-PD. The resuspended fraction containing the BMCs was filtered through a 0.45 μm syringe filter and immediately used for negative staining.

### Transmission Electron Microscopy

For transmission electron microscopy, 3 μl of enriched BMC fraction at a concentration of 20 μg/ml were mounted on carbon-coated copper grids (6 nm carbon layer, 400 mesh Grid) (Electron Microscopy Sciences, No. 456 FCF300-Cu) for 30 s and then wicked away with filter paper Whatman 50. The grids were negatively stained 3 times with 3 μl 2% (w/v) aqueous uranyl formate. Images were taken on a Tecnai 12 TEM operated at an accelerating voltage of 120 kV using an UltraScan 1000 2k × 2k CCD camera.

### Analytical Methods

Protein concentration was measured according to Bradford (Bradford, 1976). For immunological detection, BMC enriched fractions (10–20 μg) were separated on 12% polyacrylamide gels. Proteins were transferred on to a nitrocellulose membrane (Protran BA 83; GE Healthcare, United Kingdom) followed by immunoblotting with a 1:10,000 dilution of the rabbit antiserum antibodies against the shell protein PduB, PduT and internal proteins PduP and PduC as described previously (Trifunović et al., 2016). Primary antibody detection was performed with a goat anti-rabbit IgG (H + L)– horseradish peroxidase (HRP) conjugate (Bio-Rad, United States; dilution of 1:10,000).

For protein identification analysis by LC-MS/MS analysis, 30 μl on enriched BMC fractions precipitated by 5% trichloroacetic acid. Protein samples were washed twice with cold acetone, dried and dissolved in 50 μl of 10 mM Tris/HCl buffer, pH 8.2 supplemented with 0.5 μg of trypsin. The digested protein was dried and dissolved in 0.1% formic acid and following proteins were analyzed by LC-MS/MS analysis. The acquired MS data were converted to Mascot Generic File format and processed for identification using Mascot search engine (Matrix Science).

## Data Availability Statement

The raw data supporting the conclusions of this article will be made available by the authors, without undue reservation.

## Author Contributions

NPC and VM designed the study and wrote the manuscript. NPC performed, analyzed, and prepared figures. LA performed experiments. ML performed EM experiments. All authors approved the final version of the manuscript.

## Conflict of Interest

The authors declare that the research was conducted in the absence of any commercial or financial relationships that could be construed as a potential conflict of interest.

## Abbreviations

IPTG: isopropyl- β-D -1-thiogalactopyranoside
OD: optical density
PAGE: polyacrylamide gel electrophoresis
PCR: polymerase chain reaction
SDS: sodium dodecyl sulfate–polyacrylamide.
