# Supplementary material for: Exploring Bacterial Microcompartments in the Acetogenic Bacterium Acetobacterium woodii
Source: Front Microbiol. 2020 Oct 15;11:593467. doi: 10.3389/fmicb.2020.593467 (PMC7593272; doi:10.3389/fmicb.2020.593467)
Supplement: Supplementary file 1 [file Data_Sheet_1.docx]

**Supplemental Material to**

**Exploring Bacterial Microcompartments In the Acetogenic Bacterium *Acetobacterium woodii*.**

**Nilanjan Pal Chowdhury^1^, Lydia Alberti^1^, Mark Linder^2^ and Volker Müller^1*^**

*^1^Department of Molecular Microbiology & Bioenergetics, Institute of Molecular Biosciences, Johann Wolfgang Goethe University Frankfurt/Main, Max-von-Laue-Str. 9, 60438 Frankfurt, Germany*

*^2^Max Planck Institute for Biophysics, Max-von-Laue-Straße 3, D-60438 Frankfurt am Main*

**Fig. S1**. List of primers used in this study.

| Name | Sequence | Target gene |
| --- | --- | --- |
| Awo_c10540 qPCR F | GAGTGAAGTGAACGGGATCTG | Awo_c10540 |
| Awo_c10540 qPCR R | GCTGCCAAGGATGCTTCTA | Awo_c10540 |
| Awo_c19100 qPCR F | GATTTACCGGAAGGGTGTATCG | Awo_c19100 |
| Awo_c19100 qPCR R | TTAGCTGTCTTGGTAGCTTCATC | Awo_c19100 |
| Awo_c25740 qPCR F | CTAGAGGCTTGGGTGGTAAAG | Awo_c25740 |
| Awo_c25740 qPCR R | GAGACGCAATGACCGAGTAA | Awo_c25740 |
| Awo_c25840 qPCR F | GACGGGATGTCCACGATTAAA | Awo_c25840 |
| Awo_c25840 qPCR R | CCCACGACCTTTAGCACAA | Awo_c25840 |
| Awo_c25900 qPCR F | CAGTTCGTCAAGCCGTTTATG | Awo_c25900 |
| Awo_c25900 qPCR R | GAGTTGTAGTAGATGGGCAAGG | Awo_c25900 |
| Awo_c25910 qPCR F | GGTTCGTGGTGATGTTGGA | Awo_c25910 |
| Awo_c25910 qPCR R | GGTCTTGGGATTACGTGTACTG | Awo_c25910 |
| Awo_c26570 qPCR F | CAGCGCCCGTTGAAATTG | Awo_c26570 |
| Awo_c26570 qPCR R | CGAGACTGCGGCTTTAACA | Awo_c26570 |
| Awo_c28920 qPCR F | AGGCTTGAATCCCAGTGTTG | Awo_c28920 |
| Awo_c28920 qPCR R | GCCAAATCGCCCGCTATAA | Awo_c28920 |
| GyrA_for_qrtPCR | GTAAGTCGGCCCGTATTGTTG | Awo_c00060 |
| GyrA_rev_qrtPCR | AACGGATCGACCATTCCTGAG | Awo_c00060 |
| Awo_c25920Reg_For | GGGAATTCCATATGATGTACAAATTGTTTATTGTTGAAGACGAGC | Awo_c25920_Regulator |
| Awo_c25920Reg_rev | CCGCTCGAGAGAAACTTCACGCTGTTTTTCCTGAT | Awo_c25920_Regulator |
| PduA291bpUP_For | CATAAAACTAAAAAAATAGTCAAAAAAG | Upstream region of PduA |
| PduA291bpUP_Rev | AAGCTCTATCCTCCTTAATTAC | Upstream region of PduA |
| 399UPAwo25930_For_NotI | ATAGTTCACAACTTTTGGATAC | Upstream region of Awo_c25930 |
| 399UPAwo25930_Rev_NdeI | AAAACAACCCCTTTAGACAATAATC | Upstream region of Awo_c25930 |
| PduA291bpUP_For_NotI | AAGGAAAAAAGCGGCCGCCATAAAACTAAAAAAATAGTCAAAAAAG | Upstream region of PduA |
| PduA291bpUP_Rev_NdeI | GGAATTCCATATGAAGCTCTATCCTCCTTAATTAC | Upstream region of PduA |
| NPC_ermB_For | TAAAATATTCTCAAAACTTTTTAACGAGTG | Erythromycin cassette of pMTL82254 |
| NPC_ermB_Rev | CTATTAAAAATAGACAATACTTGCTCATAAGTAAC | Erythromycin cassette of pMTL82254 |

| Amino acid sequence of response regulator (Awo_c25920) with C-terminal Histidine tag |  |
| --- | --- |

**Fig. S2.** Amino acid sequence of the overproduced Awo_c25920 (response regulator) gene product from *A. woodii*. Compared to WT type, the overproduced response regulator protein contains an extra Methionine at its N-terminal and 6 extra Histidine inserted at its C-terminal for purification.

**Fig. S3.** Absolute and relative transcript abundance of BMC related genes during metabolism of fructose and 1,2-PD of *A. woodii*


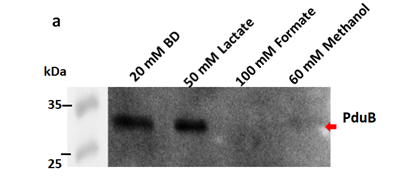


**Fig. S4**. Western blot analysis of cellular levels of PduB in cells grown of 20 mM 2,3-butanediol, 50 mM lactate, 100 mM formate and 60 mM methanol. *A. woodii* was grown on the substrates as indicated and harvested in the late exponential growth phase. Cell extracts were separated on a 12% SDS-PAGE gel. The presence of PduB was determined immunologically with antibodies raised against heterologously produced PduB
